# Manual Training in Surgery

**Published:** 1897-05-01

**Authors:** 


### Manual Training in Surgery.

The commencement of another session, when new students enter, not only into the schools, but into what is to them a new career, and when old students return to work in dissecting-room and hospital ward, seems a proper time for insisting on the importance of a detail in medical education which is somewhat apt to be lost sight of amid the many demands of the curriculum and the many terrors of the examinations, and that is the importance of everyone who wishes to attain success in surgery cultivating by every means in his power dexterity of finger and delicacy of touch. The multitude of subjects which the student of medicine now has to "get up," and the profundity of knowledge to which he has now to attain on subjects which after all are often mere matters of teaching and of memory, tend rather to lessen the importance in his eyes of that *tactus eruditus* on which the older surgeons laid so much stress. Yet, if the question received a moment's consideration, it would become apparent that now more than ever is this faculty of finger cleverness, this accurate co-ordination between muscle and nerve, necessary in the performance of surgical manipulations.

At a recent meeting of a medical society, the subject of intestinal suture being under discussion, it was said of one of the speakers that those who had seen him, while fishing, deftly and tenderly handling his flies could understand his success in the delicate manipulations necessary in some abdominal operations. There was in this something more than a mere compliment to the individual, for it expressed what we, many of us, must know to be the truth—that surgery in its higher branches is not for all. The nerve that can face the unexpected and appalling, coupled with the delicacy of touch that can mend a watch or mount a fly, or sew up an intestine, is not a common gift, yet both are wanted, and those who would excel in surgery should not hesitate to practice and develop to the utmost such gifts as they possess. Every year we see in surgery new proceedings which may, if we please, be called mere matters of technique, but on which, nevertheless, depend the success of the undertaking and the life of the patient. These are as purely matters of art and skill as is the painting of a picture; and art is long, and life is short —too often busy also—and to some it seems a waste of time to practise these little tricks, these dodges of technique. Yet labour and long practice are as necessary in this as in other arts. To know what should be done is one thing; to do it with speed and accuracy is quite another. The surgeon may be filled with knowledge and with true preception, as, indeed, may the art critic; the one as the other may know, may feel, precisely what is wanted; yet with knife or needle in his hand the surgeon may be as helpless as might the critic if in a moment of unwisdom he were to seize the brush and try to show on canvas the fancy in his mind. Sometimes this is inherent in the man, sometimes, and far more often, it is from want of education, not education of an intellectual sort, but a training of that sensibility of touch and that dexterity of muscle which enables a butcher to cut cleanly and neatly right into a joint; which enables a painter by one turn of his brush to express his idea; which enables a seamstress by a twist of finger and thumb to tie a knot on the thread with one hand; which enables an ophthalmic surgeon to remove a cataract, making it apparently creep out by itself, as if impelled by some strange magnetic force. In each case this is the artistic touch; the final product of long labour; the thing that separates the professional from the amateur; and in each case it can only be gained by practice.

We repeat, in surgery it is not enough to know what should be done. Much modern operative surgery is actually constructive. It is an art the practice of which lies outside all theory. To build up a perineum apparently out of nothing—to maintain a due proportion in the incisions, a due relation in the sutures, and to leave the newly-arranged tissues neat, firm, and durable, is a piece of artistic handicraft in which nothing but practice and training of the fingers can produce success. But in abdominal operations no one can tell what may next turn up. The mind may at any moment have to solve the most complex problems, and in proportion as the fingers can do their work almost automatically and by instinct, the mind will be left free. A Lembert's suture is a simple thing no doubt, but to make a "good job" is difficult enough, and well nigh impossible to clumsy or unaccustomed fingers. To make a joint which shall be water-tight is absolutely essential, yet in doing this no tissue must be wasted; no great pucker must be made; however distended the intestine be it must not be punctured; the stitches must be evenly distributed both on the large end and the small; and while neatness and perfection of workmanship are thought of on the one hand, time must be thought of on the other, for on time the patient's life depends. Need we then say more. Every year the actual operative work of surgery becomes more and more a matter of technique, in which trained sensitiveness of touch and trained cleverness of finger bear their part. The student, then, must not be content to know what should be done, or even to know how to do it. He must be clever not only in his mind but in his fingers; he must train and develop by practice that co-ordination of nerve and muscle which will enable him to catch a ball, to ward a blow, to mount a fly, to carve a chicken, and to mend his gloves; and beyond all these he must at every opportunity practice on the dead body the technique of his art. So much for the student. What about the surgeon? But when does the true surgeon cease to be a student? Never; and therein lies his happiness, for the true surgeon is always young and always learning.

